# Palm Kernel Cake Extracts Obtained from the Combination of Bacterial Fermentation and Enzymic Hydrolysis Promote Swine Small Intestine IPEC-J2 Cell Proliferation and Alleviate LPS-Induced Inflammation In Vitro

**DOI:** 10.3390/antiox13060682

**Published:** 2024-05-31

**Authors:** Hui Zeng, Jingna Miao, Jinghong Liao, Zhiyuan Sui, Meixin Hou, Suqin Hang

**Affiliations:** Laboratory of Gastrointestinal Microbiology, Jiangsu Key Laboratory of Gastrointestinal Nutrition and Animal Health, National Center for International Research on Animal Gut Nutrition, National Experimental Teaching Demonstration Center of Animal Science, College of Animal Science and Technology, Nanjing Agricultural University, Nanjing 210095, China; 2021105007@stu.njau.edu.cn (H.Z.); 2022805111@stu.njau.edu.cn (J.M.); 2022105022@stu.njau.edu.cn (J.L.); suizhiyuan@stu.njau.edu.cn (Z.S.); 2021105008@stu.njau.edu.cn (M.H.)

**Keywords:** proliferation, PI3K/MAPK pathway, anti-inflammatory, *CASP3*, *SLC5A1*

## Abstract

Co-fermentation with bacteria and enzymes can reduce sugar content in palm kernel cake (PKC); however, the chemical changes and their effects on cell functionality are unclear. This study investigated the active components in pre-treated PKC extracts and their effects on pig small intestine IPEC-J2 cell proliferation and LPS-induced inflammation. The extracts contained 60.75% sugar, 36.80% mannose, 1.75% polyphenols and 0.59% flavone, as determined by chemical analyses, suggesting that the extracts were palm kernel cake oligosaccharides (PKCOS). Then, we found that 1000 µg/mL PKCOS counteracted the decrease in cell viability (CCK8 kit) caused by LPS induction by 5 µg/mL LPS (*p* < 0.05). Mechanistic studies conducted by RNA-seq and qPCR analyses suggested PKCOS promoted cell proliferation through the upregulation of *TNF-α*, *PI3KAP1*, *MAP3K5* and *Fos* in the PI3K/MAPK signalling pathway; alleviated inflammation caused by LPS via the downregulation of the target genes *Casp3* and *TNF-α* in association with apoptosis; and regulated the expression of the antioxidant genes *SOD1*, *SOD2* and *GPX4* to exert positive antioxidant effects (*p* < 0.05). Furthermore, PKCOS upregulated *SLC5A1* (encoding SLGT1), *HK* and *MPI* in the glycolytic pathway (*p* < 0.05), suggesting cell survival. In summary, PKCOS has positive effects on promoting swine intestine cell proliferation against inflammation.

## 1. Introduction

Palm kernel cake (PKC), a by-product of the mannan-rich palm oil industry [1], has received close attention in recent years as an unconventional feed resource. Treatment with enzymes and microorganisms can increase the nutritional value of PKC, primarily by reducing the amount of cellulose and mannan [2]. Numerous active substances are produced during the process, including volatile organic compounds (VOCs), such as ketones, aromatic hydrocarbons and short-chain fatty acids (SCFAs); microbial metabolites, such as amino acids, enzymes and antibiotics [3,4]; and oligosaccharides [5], thus promoting PKCs’ use in livestock, particularly in monogastric animals which cannot secrete various cellulases. Oligosaccharides are short-chain polymers with a low molecular weight, and they consist of 2–10 monosaccharide units [6] and present specific biological activities, such as aiding in the proliferation of Bifidobacteria, the regulation of gastrointestinal function, anti-inflammatory responses and the reduction in disease [7,8].

Free radicals can be produced in many situations, such as physical conditions, chemical reactions and metabolic processes. When the body is subjected to an inflammatory stimulus, a significant increase in the production of free radicals occurs, resulting in their accumulation. The hydroxyl radical, one of the reactive oxygen species, is commonly formed in vivo and can cause serious damage to biomolecules, such as lipids, proteins and nucleic acids [9]. This damage can lead to inflammation-related diseases, such as inflammatory bowel disease (IBD) and bacterial diarrhoea, particularly severe diarrhoea, in weaned piglets [10]. Saccharides, which originate either from synthetic chemicals [11] or natural substances [12], were revealed to have strong radical scavenging abilities [13]. Natural antioxidants from organism extracts have recently received increased interest due to consumer concerns.

The small intestine is not only the main compartment of digestion and absorption but is also the first physical barrier against external factors. Pathogenic microorganisms, harsh environmental sanitation (such as unsuitable temperature and humidity) and weaning stress often cause reduced immunity and intestinal structural abnormalities, leading to dysfunction in piglets [14]. Lipopolysaccharide (LPS) is a major culprit in porcine intestinal injury and is associated with inflammation, apoptosis, proliferation, differentiation and host immune activation [15]. Therefore, gut health is crucial to maintaining the integrity of small intestine functions and host health [16,17]. Porcine epithelial cells (IPECs) are the first barrier against exogenous antigens, pathogens and toxins entering the circulatory system, including cyto-inflammatory factors, such as tumour necrosis factor-α (*TNF-α*) and interleukin-6 (*IL-6*) [18]. The IPEC-J2 cell line, well-known as the swine jejunal epithelial cell line, is isolated from newborn piglets and is widely used for studying gut inflammation responses, immunity and barrier integrity in vitro [19,20]. A study showed that chitosan oligosaccharides could attenuate LPS-induced inflammation in IPEC-J2 cells by modulating the TLR4/NF-κB signalling pathway [21]. However, it is worth noting that cellular life processes, particularly cell proliferation and anti-inflammation, are linked to glycolysis [22]. For example, cell proliferation and differentiation, free radical scavenging and autophagy all require energy [23,24].

Our previous study demonstrated that the use of bacterial fermentation integration with complex enzymatic hydrolysis effectively degraded cellulose in PKC and produced a large amount of reducing sugars [25], which may be rich in potentially positive functional substances, such as oligosaccharides [26]. However, studies focused on extracts, mainly oligosaccharides derived from PKC and their potential effects, particularly on gut intestinal health, are rare and have yet to be further explored. Our hypothesis is that palm kernel cake oligosaccharides (PKCOS) can enter the cell via glucose transporters and have a positive impact on the life process of IPEC-J2. In this study, PKCOS was first extracted from pre-treated PKC and then partially characterised to evaluate its antioxidant activity. Finally, the effects of PKCOS on gut epithelial functions using the IPEC-J2 cell line as a model were investigated. This study will contribute to the development of novel multifunctional substances that can strengthen the health of animal gut intestines.

## 2. Materials and Methods

### 2.1. Materials and Chemicals

Lipopolysaccharide (LPS), purchased from Yuanye Lot., Shanghai, China, was prepared by dissolution in phosphate buffer saline (PBS) purchased from Solaibold Lot., Beijing, China, and then stored at −20 °C. Foetal bovine serum (FBS) was purchased from Tianhang Lot. (Huzhou, China). TRIzol was purchased from Biosharp Lot. (Beijing, China). Gene sequences were designed by Accurate Biology Lot. (Changsha, China) and purchased from Qingke Lot. (Nanjing, China). All the primer sequences are shown in Table 1. Other reagents were purchased from Solaibold Lot. (Beijing, China), such as dimethyl sulfoxide (DMSO) and Dulbecco’s Modified Eagle Medium/Nutrient Mixture F-12 (DMEM/F12).

### 2.2. Preparation of PKCOS and Content Analysis

According to the description of the previous study by Xiang [27], slight modifications were made. In brief, pre-treated PKC were extracted twice using 50% ethanol at a ratio of 1:20 (PKC to ethanol, m/v) for 1 h at 60 °C. The extracted solution was centrifuged at 4000 rpm/min for 5 min, and then the supernatant was freeze-dried (LGJ-10, Deyang Yibang Lot., Shanghai, China). Finally, the freeze-dried powder was dissolved in DMSO to prepare a stock solution of 1000 µg/mL for cell assays or a stock solution of PBS 20 mg/mL for chemical analysis including total sugars, mannose and polyphenols. Everything was stored at −20 °C.

The total sugars and proteins were determined using the phenol–sulphuric acid colorimetric method [28] and Coomassie Bright Blue (Beyotime Lot., Beijing, China), respectively. Mannose was analysed using the procedure reported by Qi [29]. The determination of polyphenols and flavonoids referred to Adom’s method [30], and were denoted as rutin and gallic acid equivalents (mg/g PKCOS).

### 2.3. In Vitro Antioxidant Evaluation of PKCOS

Total antioxidant capacity (T-AOC), hydroxyl radical (^•^OH) inhibition rate and 1,1-diphenyl-2-picryl-hydrazyl radical scavenging rate (^•^DPPH) were all measured according to the kit instructions (Jiancheng, Lot., Nanjing, China).

### 2.4. IPEC-J2 Cell Line and Culture In Vitro

IPEC-J2 cells were donated by Weiyun Zhu, a faculty member at Nanjing Agricultural University. The thawed IPEC-J2 cells were first seeded in T75 cell culture dishes (Corning Lot., Corning, NY, USA) and then cultured in DMEM/F12 supplemented with 10% FBS and 1% penicillin–streptomycin at 37 °C in humidified air containing 5% CO_2_ until the cell density reached nearly 80%. The cells were passaged for the following experiments.

### 2.5. Effects of PKCOS on Cell Viability and Underlying Mechanisms

#### 2.5.1. Effects of PKCOS and LPS on IPEC-J2 Cell Viability

The IPEC-J2 cells were seeded in 96-well plates (Corning Lot., Corning, NY, USA) at a density of 4 × 10^4^ cells/mL in the culture medium overnight. Then, the cells were treated by either 0, 1, 20, 50, 100, 250, 500 or 1000 µg/mL PKCOS contained in DMEM/F12 medium for 6 h or treated by 0, 0.1, 1, 5, 10 or 20 µg/mL LPS contained in DMEM/F12 medium for 12 h, respectively. In the end, the cell survival rate was detected. The trial was repeated for three batches, each containing 6 replicates. Then, appropriate concentrations of PKCOS and LPS were obtained based on survival for further studies.

The cells were treated with the appropriate concentration of LPS (5 µg/mL) for 12 h, and then the appropriate concentration of PKCOS (0, 250, 500 µg/mL or 1000 µg/mL) was added in each well and cultured for 6 h to determine whether PKCOS could alleviate the damage induced by LPS on IPEC-J2. Similarly, cell viability was determined at the end of the culture, and the trial was repeated for three batches, with 6 replicates in each batch.

#### 2.5.2. Mechanistic Exploration of PKCOS’s Effects on the Survival Rate of IPEC-J2 Cells with or without LPS

To explore the underlying mechanisms of the effects of PKCOS on cell survival rate with or without LPS, cells were cultured in 10 cm cell culture dishes (Corning, USA) and divided into five groups, namely, the control group, named CT, treated by PBS; the DMSO group, treated by DMSO (PKCOS was dissolved in DMSO); the PKCOS group, treated by the appropriate concentration of PKCOS selected from above (1000 µg/mL) for 6 h; the LPS group, treated by the selected appropriate concentration of LPS above (5 µg/mL) for 12 h; and the LPS-PKCOS group, treated by LPS for 12 h and then supplemented with PKCOS for 6 h. Finally, cells from the five treatments were collected, and the RNA was extracted by TRIzol reagent and then stored at −80 °C for future RNA-seq and qPCR analyses. Three replicates were performed in each group/treatment (*n* = 3).

### 2.6. Cell Survival Rate Assay

The cell survival rate was determined via the CCK-8 assay according to the manufacturer’s instructions (Baiscience Lot., Beijing, China) and expressed as a viability percentage according to the following formula:

$$\mathrm{Cell}\;\mathrm{survival}\;\mathrm{rate}\;\left(\%\right)=\frac{\mathrm{absorbance}\;\mathrm{of}\;\mathrm{treatment}-\;\mathrm{absorbance}\;\mathrm{of}\;\mathrm{blank}}{\mathrm{absorbance}\;\mathrm{of}\;\mathrm{control}-\;\mathrm{absorbance}\;\mathrm{of}\;\mathrm{blank}}\;\text{×}\;100\%$$

### 2.7. Bioinformatics Analysis and qPCR

According to a previous study [31], the procedure of RNA extraction from the IPEC-J2 cells was performed based on the manufacturer’s instructions (Invitrogen, Waltham, MA, USA), and genomic DNA was removed using DNase I (TaKara. Lot., Beijing, China). RNA quality was tested using a 2100 Bioanalyzer (Agilent, Santa Clara, CA, USA) and quantified by ND-2000 NanoDrop (Thermo Scientific, Wilmington, DE, USA), respectively. Subsequently, cDNA libraries were created following the instructions of the TruSeqTM RNA sample preparation kit (RS-122-2101, Illumina, San Diego, CA, USA), and sequencing was performed via Illumina NovaSeq 6000 sequencing (BIOZERON Co. Ltd., Shanghai, China). A *p*-value < 0.05 was used as a criterion to identify differentially expressed genes (DEGs). Kyoto Encyclopedia of Genes and Genomes (KEGG) enrichment analyses were performed using Kobas (http://Kobas.cbi.pku.edu.cn/home.do, accessed on 15 November 2023).

All samples were kept at a constant RNA concentration of 500 ng/μL using enzyme-free water. qPCR was conducted using the SYBR Green Premix Pro Taq HS qPCR Kit (AG11718, Agbio Lot., Changsha, China) on a QuantantStudio7 Flex (ABI). The reaction system for qPCR consisted of 20 μL, with a template addition of 20 ng. The qPCR procedure was as follows: Step 1: 95 °C for 30 s, repeated 1 time; Step 2: 95 °C for 5 s, followed by 60 °C for 30 s, repeated 40 times. The primer sequences used are listed in Table 1, showing that the antioxidant gene primers are consistent with those reported in the literature [32,33]. Finally, using the 2^−ΔΔCq^ method, the relative expression of the target genes was assessed.

**Table 1:** Real-time fluorescence quantification of gene sequences and primer information.

Gene	Version	Forward Primer (5′-3′)	Reverse Primer (5′-3′)
*β-actin*	XM_021086047.1	AACTACCTTCAACTCCATCAT	GATCTCCTTCTGCATCCTGT
*TNF-α*	NM_214022.1	CACGCTCTTCTGCCTACTGC	ACGATGATCTGAGTCCTTGG
*IL-6*	NM_214399.1	GCTTCTGGTGATGGCTACTG	GCCGAGGATGTACTTAATGAGTTC
*PIK3AP1*	NM_001244503.1	TCCAGCACAGCAAGCAG	GTAACCTCGGGCACTTCATT
*CASP3*	NM_214131.1	GGACTGCTGTAGAACTCTAACTGG	CAAGAAGTCTGCCTCAACTGGTA
*CMKLR1*	NM_001123100.1	CAAGAAAGAAGTCGGGGAACAC	CTCTGGGAGCTGGCTGTGAT
*FOS*	NM_001123113.1	CCCCAGAAGAAGAAGAGAAAAGG	GTCTGTCTCCGCTTGGAGTGT
*SOCS3*	NM_001123196.1	ATCCCTCTGGTGTTGAGCCG	GCCGTTGACTGTTTTCCGAC
*MAP3K5*	XM_021072505.1	CACCGGGATATAAAGGGTGACAA	CAAATGTCTGCTGCCTTCCC
*BCL2L1*	XM_021077294.1	GTGAACTGGGGTCGCATTGT	CCTTGTCTACGCTCTCCACG
*LIF*	NM_214402.2	TGTACCGCATCATCGCCTAC	CAGGTTCACAGCACCAGGAT
*SLC5A1*	XM_021072101.1	TCATCATCGTCCTGGTCGTCTCC	TGAATGTCCTCCTCCTCTGCATCC
*SLC2A2*	NM_001097417.1	TGCTCTGGTCTCTGTCTGTGTCC	ATTCTTCCAAGCCGATCTCCAAGC
*MPI*	NM_001253921.1	ACCTTTCTGACGAAGGTGCC	AGATCCGCTTCACCAACAGG
*PMM2*	XM_021086654.1	CCGAAACGGGATGTTGAACG	GCTGATCTGGCCTCCTATGG
*SOD1*	NM_214201.1	CGTGCAACCAGTTTGGACAT	AGCATGAAGTTGGGCTCGAA
*SOD2*	NM_214127.2	CTTGCAGATTGCCGCTTGTT	CGGCGTATCGCTCAGTTACA
*GPX1*	XM_021081498.1	TGTACCCGCTATTCTGGGGA	TCACACAGGCGTTTCCTCTC
*GPX4*	NM_001190422.1	AACCAGATGACTTGGGCAGA	AGACCATGGCATGAGGGAAT
*CAT*	NM_214407.1	TGTGGTTTACGGATTCTGG	CCTTGGGCTGGACTTTCA
*NRF2*	XM_005654811.3	TACATGCACTTTGGGGAGGT	AGATCGTCCCGGCTAATGAG
*KEAP1*	XM_021075132.1	AGAGCCCAGTCTTCATTGCT	TGTCCTGTTGCATACCGTCT

### 2.8. Statistical Analysis

The data were expressed as the mean ± standard deviation (SD). The results of figures were analysed via one-way analysis of variance (ANOVA) using SPSS 25.0 software and plotted using GraphPad Prism 8.0.2. The volcano plots of DEGs were plotted by http://www.biozeron.com, and the KEGG pathway enrichment analyses of DEGs were conducted on https://www.omicstudio.cn/. A *p*-value less than 0.05 indicated statistical significance.

## 3. Results

### 3.1. Contents of PKCOS

As shown in Table 2, the acquisition rate of PKCOS is nearly 9.62%, and they are mainly composed of sugars and mannose, accounting for 60.75% and 36.80%, respectively, and a very small amount of protein, which accounts for nearly 0.19%. It also contains small amounts of polyphenols and flavonoids, the values of which are 17.30 ± 0.02 (gallic acid equivalents) mg/g PKCOS and 5.90 ± 0.01 (rutin equivalent) mg/g PKCOS, respectively.

### 3.2. Evaluation of Antioxidant Activity of PKCOS In Vitro

PKCOS (20 mg/mL) demonstrates certain antioxidant activity in vitro, in which the total antioxidant capacity (T-AOC) reached 0.296 ± 0.007 mM Trolox, the hydroxyl (^•^OH) radical scavenging rate reached 419.14 ± 5.76 U/mL, equating to an inhibition percentage of 53.91 ± 1.62%, and the ^•^DPPH radical scavenging rate reached 2934.41 ± 13.14%.

### 3.3. Effect of PKCOS or LPS on IPEC-J2 Cell Viability

**Figure 1:**
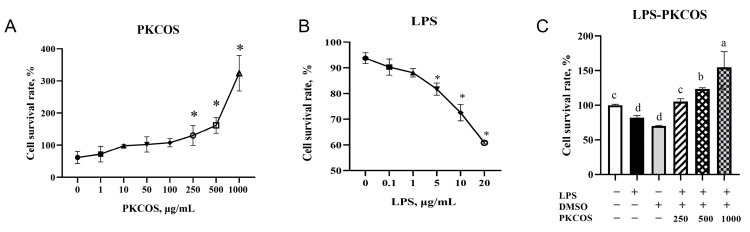
Cell survival rate of IPEC-J2 cells with different treatments. (**A**) Cell survival rate of different concentrations of PKCOS on the viability of IPEC-J2 cells. (**B**) Cell survival rate of different concentrations of LPS on the viability of IPEC-J2 cells. (**C**) IPEC-J2 cells were treated with PBS or 5 µg/mL LPS at the indicated concentrations for 24 h and then treated with 250, 500 or 1000 µg/mL PKCOS. Data are presented as mean ± SD, and the experiment consisted of 3 batches, with each batch containing 6 replicates. Bars without a common letter (a,b,c,d) and ‘*’ denote statistically significant differences (*p* < 0.05).

IPEC-J2 cell survival rates were increased in a dose-dependent pattern by the different concentrations of PKCOS treatment, particularly at the concentrations of 250, 500 and 1000 µg/mL PKCOS (Figure 1A, *p* < 0.05). In contrast, IPEC-J2 cell viability induced by LPS in a range of 5 to 20 µg/mL presented a dose-dependent reduction (Figure 1B, *p* < 0.05), indicating that the cells could be damaged by LPS, and the appropriate concentration is 5 µg/mL. Figure 1C shows that PKCOS alleviates LPS-induced (5 µg/mL) cell apoptosis (*p* < 0.05); in particular, the addition of 250 µg/mL PKCOS returned the value of cell viability to normal, while the 1000 µg/mL concentration of PKCOS improved cell viability by around 150% (*p* < 0.05).

### 3.4. PKCOS Enhanced Cell Viability and Altered Gene Transcription Expression

**Figure 2:**
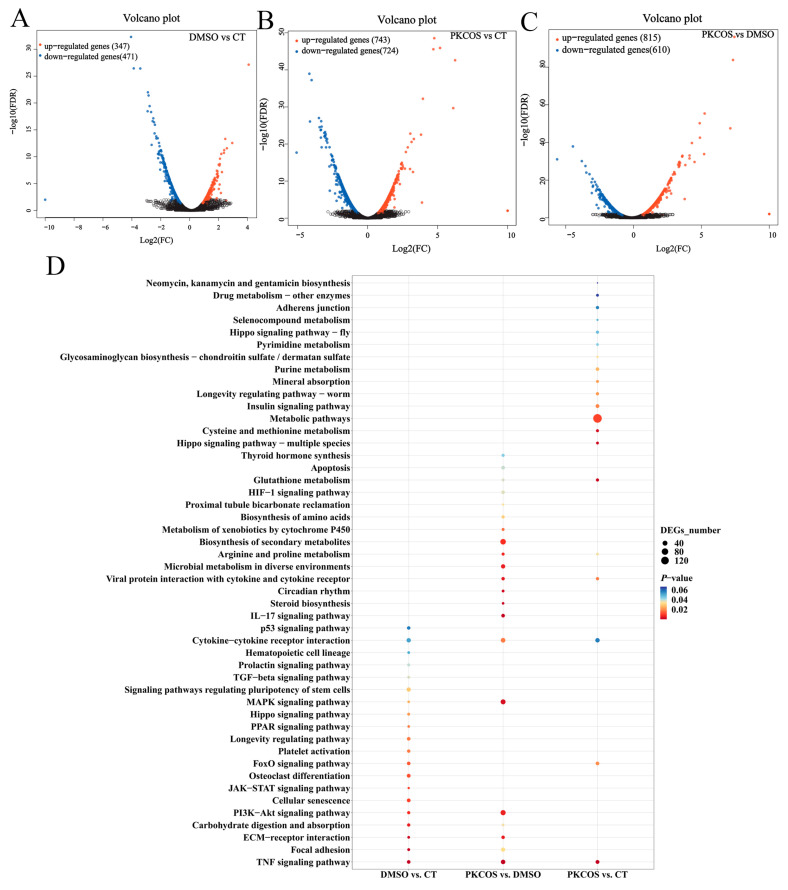
PKCOS vs. DMSO vs. CT transcriptome analysis. (**A**) Volcano plot of DEGs in the DMSO and CT groups. (**B**) Volcano plot of DEGs in the PKCOS and CT groups. (**C**) Volcano plot of DEGs in the PKCOS and DMSO groups. Upregulated genes are shown in red, downregulated genes are shown in blue and genes with no significant difference in expression are indicated in black; *p* < 0.05 indicates significance. (**D**) KEGG pathway enrichment analysis of DEGs in the DMSO and CT groups, the PKCOS and DMSO groups and the PKCOS and CT groups. The size and colour of the circles represent the number of enriched genes and the *p*-value; *n* = 3.

A total of 347 upregulated and 471 downregulated DEGs were identified in the DMSO group, and 743 upregulated and 724 downregulated DEGs were identified in the PKCOS group compared with those identified in the CT group (Figure 2A,B), while 815 upregulated and 610 downregulated DEGs were identified in the PKCOS group compared with those identified in the DMSO group (Figure 2C). Furthermore, a series of signalling pathways were enriched by KEGG pathway analysis of DEGs, such as the metabolism of cell proliferation, including the MAPK signalling pathway, the PI3K-Akt signalling pathway, the TNF signalling pathway and cytokine–cytokine receptor interaction (Figure 2D). Therefore, qRT-PCR was performed to confirm that the above pathways were related to the metabolism of cell proliferation.

**Figure 3:**
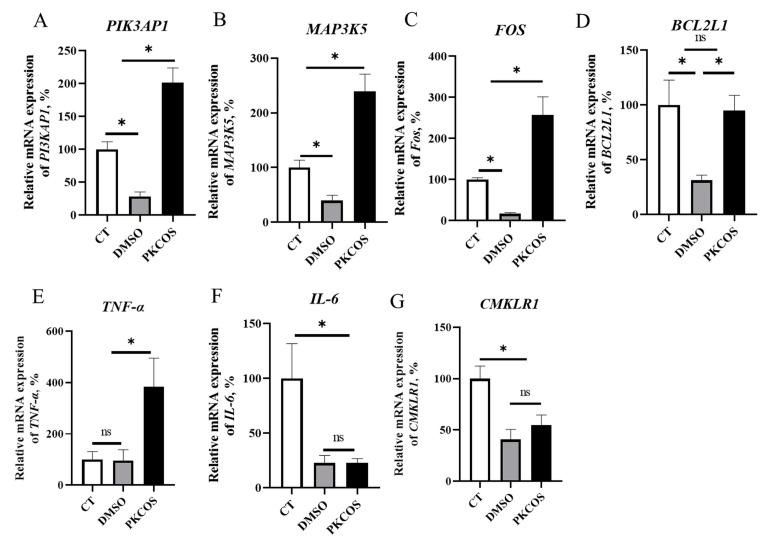
Real-time PCR verified the key genes in the three groups of KEGG-enrichment pathways (PKCOS vs. DMSO vs. CT). The relative mRNA expressions of *PI3KAP1*, *MAP3K5*, *FOS*, *BCL2L1*, *TNF-α*, *IL-6*, *CMKLR1* among the CT, DMSO, PKCOS groups (**A**–**G**). “*” indicates a statistically significant difference, *p* < 0.05, while “ns” indicates no statistical difference. The data are expressed as mean ± SD; *n* = 3.

Compared with the CT treatment, DMSO showed a trend towards the decreased mRNA expression of genes *PI3KAP1*, *MAP3K5*, *Fos* and *BCL2L1* (Figure 3A–D, *p* < 0.05), while PKCOS showed a trend towards the increased expression of genes *PI3KAP1*, *MAP3K5* and *Fos* and the restored mRNA expression of *BCL2L1* (*p* < 0.05). Compared with the CT and DMSO groups, PKCOS enhanced the expression of cytokine TNF-α (Figure 3E, *p* < 0.05), while the expressions of cytokine IL-6 in both the DMSO group and the PKCOS group were lower than that in the CT group (Figure 3F, *p* < 0.05). Similar to the IL-6 expressions, the expression of CMKLR1, a member of the G protein-coupled receptor (GPCR) family, demonstrated lower values in both the DMSO and PKCOS groups than in the CT group (Figure 3G, *p* < 0.05).

### 3.5. PKCOS Attenuated IPEC-J2 Cell Apoptosis Induced by LPS

**Figure 4:**
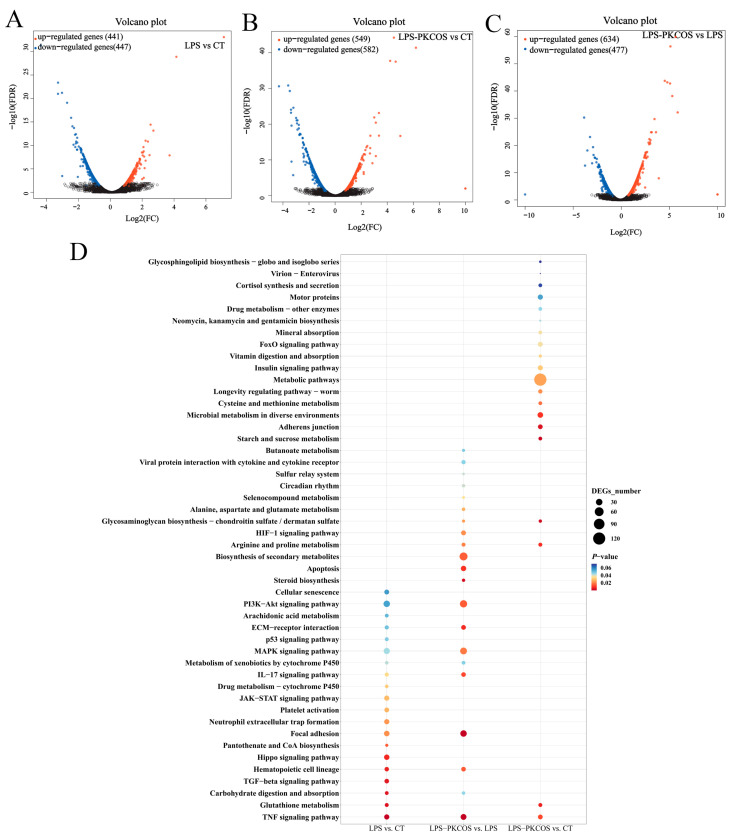
LPS-PKCOS vs. LPS vs. CT transcriptome analysis. (**A**) Volcano plot of DEGs in the LPS and CT groups. (**B**) Volcano plot of DEGs in the LPS-PKCOS and CT groups. (**C**) Volcano plot of DEGs in the LPS-PKCOS and LPS groups. Upregulated genes are shown in red, downregulated genes are shown in blue and genes with no significant difference in expression are indicated in black; *p* < 0.05 indicated significance. KEGG pathway enrichment analysis of DEGs in the LPS and CT groups, the LPS-PKCOS and LPS groups and the LPS-PKCOS and CT groups. (**D**) The size and colour of the circles represent the number of enriched genes and the *p*-value; *n* = 3.

In total, 441 upregulated and 447 downregulated DEGs were identified in the LPS group (Figure 4A), and 549 upregulated and 582 downregulated DEGs were identified in the LPS-PKCOS group compared with the CT group (Figure 4B), while 634 upregulated and 477 downregulated DEGs were identified in the LPS-PKCOS group compared with the LPS group (Figure 4C). The predominantly enriched pathways based on a KEGG pathway analysis of DEGs were mainly associated with cell apoptosis, including the IL-17 signalling pathway, the PI3K-Akt signalling pathway, the TNF signalling pathway, focal adhesion and ECM–receptor interaction (Figure 4D). Similar to the above, qPCR was performed to confirm those predominantly enriched pathways.

**Figure 5:**
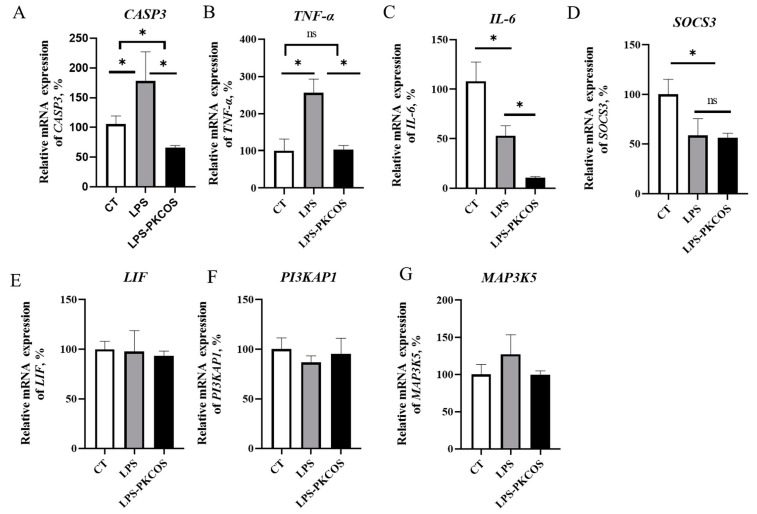
Real-time PCR verified the key genes in the three groups of KEGG-enrichment pathways (LPS-PKCOS vs. LPS vs. CT). The relative mRNA expressions of *CASP3*, *TNF-α*, *IL-6*, *SOCS3*, *LIF*, *PI3KAP1*, *MAP3K5* among the CT, LPS, LPSS-PKCOS groups (**A**–**G**). “*” indicates a statistically significant difference, *p* < 0.05 while “ns” indicates no statistical difference. The data are expressed as mean ± SD; *n* = 3.

The qPCR results showed that the expression of genes *CASP3* and *TNF-α* was increased in the LPS group compared with the CT group (Figure 5A,B, *p* < 0.05); however, the expression of two genes in LPS-PKCOS decreased to the value close to that in the CT group. A significant difference in *IL-6* expression was observed among the three groups (CT, LPS and LPS-PKCOS; Figure 5C, *p* < 0.05), and it was slightly lower in the LPS group and the lowest in the LPS-PKCOS group compared to the CT group. The expression of *SOCS3*, a major regulator of infection and inflammation associated with *IL-6* and *LIF*, was observed to have lower values in both the LPS and LPS-PKCOS groups than in the CT group, and no difference was revealed between the LPS and LPS-PKCOS groups (Figure 5D), while the expression of the *LIF* gene showed no difference among the three groups (CT, LPS and LPS-PKCOS; Figure 5E). Similarly, the expressions of the genes *PI3KAP1* and *MAP3K5* in the PI3K/MAPK signalling pathways showed no differences across the three groups (Figure 5F,G).

### 3.6. PKCOS Promotes the Glycolysis of Cells via HK and MPI Enzymes

**Figure 6:**
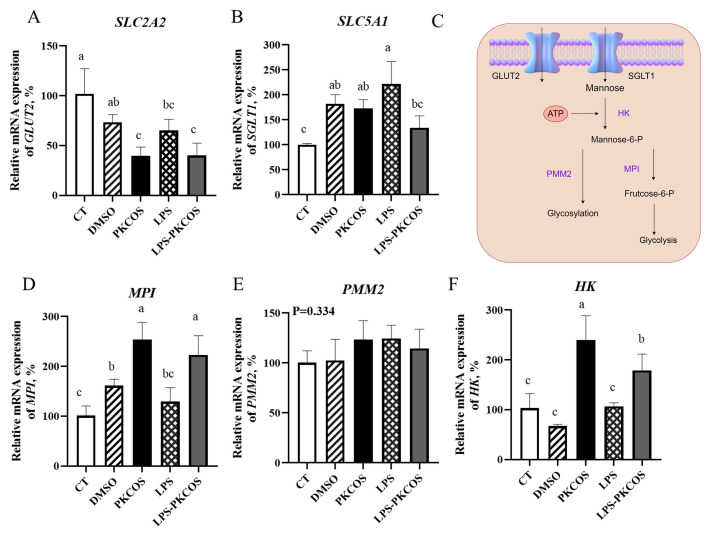
Possible glucose transporters of the cellular uptake of mannose and the gene expression of three key enzymes involved in mannose metabolism. Relative mRNA expression of SLC2A2 and SLC5A1 (**A**,**B**). GLUT2, glucose transporter protein 2, edited by SLC2A2; SGLT1, Na+/Glucose Cotransporter 1, edited by SLC5A1. Schematic diagram of the mannose metabolism pathway (**C**). Relative mRNA expression of genes for key enzymes in mannose metabolism (**D**–**F**). *MPI*, the gene for mannose phosphate isomerase; *PMM2*, the gene for phosphomannomutase 2; *HK*, the gene for hexokinase. Data are presented as mean ± SD (*n* = 3). Bars without a common letter (a,b,c) denote statistically significant differences (*p* < 0.05).

Based on the significant amount of mannose contained in PKCOS, the expression of genes *SLC2A2* and *SLC5A1*, encoding the glucose transporters GLUT2 and SGLT1, respectively, was paid close attention. The *SLC2A2* expression detected by qPCR in the DMSO group was close to the value in the CT group, while its expression in the other three groups (PKCOS, LPS and LPS-PKCOS) was reduced (*p* < 0.05). There was no difference in the relative mRNA expression of *SLC2A2* between the PKCOS and LPS-PKCOS groups, and the values in both groups were the lowest (Figure 6A, *p* < 0.05). In contrast, the transcriptional expressions of *SLC5A1* increased in the other four groups compared to the CT group. Interestingly, the transcription of *SLC5A1* increased in the LPS group and decreased in the LPS-PKCOS group (Figure 6B, *p* < 0.05). Regarding the enzymes involved in mannose metabolism (Figure 6C), the gene mRNA expressions of three key enzymes—*HK*, *MPI* and *PMM2*—were evaluated by qPCR. The mRNA expression of *HK* and *MPI* in the PKCOS and LPS-PKCOS groups exhibited an increase compared with that in the CT group (Figure 6D,F, *p* < 0.05). However, the mRNA expressions of *PMM2* showed no difference among any of the five treatments (Figure 6E).

### 3.7. PKCOS Readjusted the Antioxidant Genes’ mRNA Expressions

**Figure 7:**
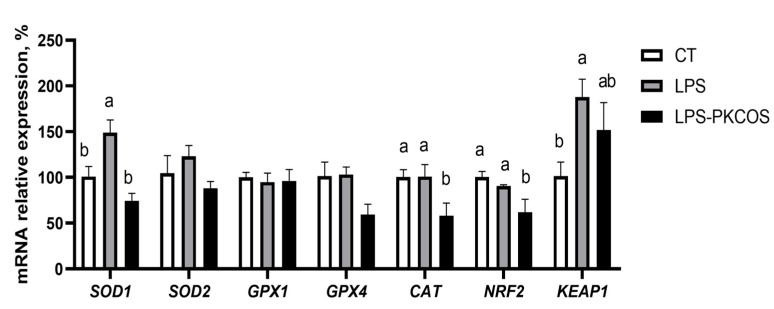
Relative mRNA expression of antioxidant genes, including *SOD1*, *SOD2*, *GPX1*, *GPX4*, *CAT*, *NRF2* and *KEAP1*. Data are presented as mean ± SD (*n* = 3). Bars without a common letter (a,b) denote statistically significant differences (*p* < 0.05).

Given that the PKCOS extract demonstrated antioxidant properties in an in vitro antioxidant assay, we postulated that PKCOS may also modulate oxidative stress in the LPS model of inflammation. Consequently, we examined the expression of antioxidant genes among the CT, LPS and LPS-PKCOS groups. As a result, PKCOS downgraded the trend of high levels of *SOD1*, *SOD2* and *KEAP1* caused by LPS, upgraded the tendency of low levels of GPX4 caused by LPS and decreased *CAT* and *NRF2* mRNA expression (Figure 7, *p* < 0.05); it had no effect on *GPX1*.

## 4. Discussion

Our study showed that PKCOS extracted by 50% alcohol is composed of sugar, particularly mannose, and a very small amount of phenols, flavonoids and proteins. The explanation for this may be attributed to sugar also being able to bind covalently to other molecules, such as phenols [34]. It has been previously reported that 50% ethanol is an effective solvent for the extraction of oligosaccharides [35], and polysaccharides are not soluble in such high concentrations of ethanol [36]. Therefore, it is speculated that oligosaccharides are the major components of PKCOS. The ^•^OH and ^•^DPPH radicals are an extremely active free radical in biological systems and can cause oxidative damage to DNA [37]. In our study, PKCOS exhibited higher T-AOC activity than açai fermentation extracts [38] but lower than Buddleja scordioides Kunth [39], higher ^•^DPPH radical scavenging activity in vitro than BHA [40] and higher ^•^OH radical percentage of inhibition than gallic acid [41], which may indicate that PKCOS acts as a potential antioxidant agent.

Cell activity and proliferation are frequently associated with sugar levels and their metabolism [42]. In the present study, 1000 µg/mL PKCOS demonstrated an enhancement of IPEC-J2 cell viability, which is consistent with previous findings of oligosaccharides in promoting intestinal cell proliferation in piglets [43] and periodontal ligament stem cell proliferation in humans [44]. The results of the gene expression conducted by qPCR demonstrated a higher expression of *TNF-α*, *PI3KAP1*, *MAP3K5* and *FOS* in the PKCOS group than in the CT group. *TNF-α* is an upstream transcriptional regulator, while *Fos* and *Bcl2L1* are downstream transcriptional regulators in PI3K/MAPK signalling pathways [45]. c-Fos (edited by *Fos*) is a nucleophosphoprotein that heterodimerises with c-Jun and then forms an AP-1 (activator-1) complex, which binds to DNA at specific sites in the promoter and enhancer regions of the target gene and takes on the role of signal transduction [46]. Studies have shown that the activation of the PI3K/MAPK pathway promotes the proliferation of zebrafish subintestinal vascular epithelial cells [47] and restoration of platelet function [48]. Therefore, PKCOS may play an important role in promoting IPEC-J2 cell proliferation, and the promotion was performed through the PI3K/MAPK signalling pathway. Bcl2L1 (Bcl-XL), belonging to the Bcl-2 family, is the main antiapoptotic protein [49,50], and its mRNA expressions in the CT and PKCOS groups were similar, implying that there was no damage to IPEC-J2 cell viability treated by PKCOS. Ultimately, the pro-cellular proliferative mechanisms of PKCOS can be summarised as upregulating the expression of *TNF-α*, *PI3KAP1*, *MAP3K5* and *Fos* in PI3K/AMPK signalling pathways.

From the metric of cell viability, it was clear that the 1000 µg/mL concentration of PKCOS was the applicable dose for relieving LPS-induced damage. Further investigation of the potential pathway by which it alleviated LPS-induced cell inflammation and apoptosis may indicate the involvement of the IL-17/TNF-α signalling pathway. Programmed cell death (PCD) is the process in which cells eliminate themselves in a controlled manner [51]. Caspases (particularly caspase-3) are mediators of the signalling pathways for apoptosis and cell breakdown and play a role in chromatin condensation and DNA degradation during apoptosis [52,53]. *TNF-α* acts as a signalling molecule in the inflammatory response [54]. Increased levels of *CASP-3* and *TNF-α* were observed in this study, which is in agreement with a previous study that APAP-induced inflammation in HepG2 Cells [55], suggesting that TNF-α and IL-17 signalling pathways were significantly enhanced by LPS treatment, based on findings from transcriptome analysis, which may be associated with cellular inflammatory and immune processes [56]. LPS-induced intestinal injury is usually characterised by the overproduction of inflammatory cytokines, such as TNF-α, iNOS and IL-1β. Chitosan oligosaccharides can block LPS-induced inflammatory responses and reduce the death of cells [57]. As expected, the levels of *CASP-3* and *TNF-α* mRNA were significantly decreased after PKCOS treatment, so that the cell survival rate of IPEC-J2 was significantly increased. These results suggest that the regulation of PKCOS on the mRNA expression of *TNF-α* and *CASP3* may result from multi-target genes and play a protective role in the LPS-induced apoptosis of IPEC cells. Similar to the other oligosaccharides obtained from plant/food/feed reported by other researchers [58], PKCOS presented anti-inflammatory effects through downregulating the expressions of target genes associated with cell death, such as *CASP3* and *TNF-α*.

In addition, LPS caused high *SOD1* and *SOD2* levels in the IPEC-J2 cells, while PKCOS restored levels of *SOD1* and *SOD2* nearly to that in the CT group. High *SOD1* and *SOD2* levels were main indicators of cellular inflammation and oxidative stress [59,60,61]. This suggests that PKCOS can alleviate LPS-induced oxidative stress and inflammatory responses in IPEC-J2 cells. Increased *GPX4* may inhibit the onset of cellular iron death [62,63]. It is noteworthy that our study showed that PKCOS can reverse the downregulation of *GPX4* induced by LPS. *CAT* was also a closely related indicator of cell death [64], and the qPCR results showed that PKCOS downregulated the mRNA expression of this gene. This indicates that the active substances in the feed have antioxidant capacity, likely through the NRF2/KEAP1 pathway [65]. In our study, PKCOS was known to downregulate the expression of *NRF2* and normalise the expression of *KEAP1*. These alterations in antioxidant genes indicated that PKCOS also served as an antioxidant in the LPS-induced IPEC-J2 inflammation model. Considering that PKCOS has a role in regulating the expression of antioxidant genes, we concluded that PKCOS has antioxidant and anti-inflammatory capacities.

Sugar metabolism is a determinant of cell death and cell proliferation [66]. Thus, the question is whether PKCOS affects the glycolytic pathway during IPEC-J2 proliferation and apoptosis. Mannose, an isoform of glucose, generally enters cells through glucose transporters. Therefore, a new question of whether mannose in PKCOS can enter cells through glucose transporters is posed. SGLT1 and GLUT2 are the main glucose transporters in the gut [67,68]. The high level of *SLC5A1* mRNA expression and the tendency towards a low mRNA expression level of *SLC2A2* in the PKCOS and LPS-PKCOS groups compared to those in the CT group suggest that mannose in PKCOS may enter cells through SGLT1. The expression pattern of *SLC5A1* in LPS-PKCOS was consistent with previous findings, in which stevia leaf extracts promoted the activity and expression of SGLT1 and enhanced the intestinal capacity to absorb glucose in rabbits [69]. After entering into cells, mannose can be catalysed by hexokinase (HK) to produce 6-phospho-mannose (M6P), which is then catalysed by phosphomannose isomerase (MPI) into the glycolytic pathway or catalysed by phosphomannose mutase (PMM2) into the glycosylation pathway [70,71]. Interestingly, the transcriptional expression of gene *HK*/*MPI* was increased by PKCOS treatment with and without added LPS, while the mRNA expression of *PMM2* did not show a difference across the five groups, suggesting that PKCOS promoted the glycolytic pathway in IPEC-J2 cells, which may be associated with cell proliferation and alleviation of inflammation-induced apoptosis [72]. Thus, one possible regulatory pathway is that PKCOS may enter cells through the transporter SGLT1 and promote the glycolytic pathway associated with cell proliferation and the alleviation of inflammation-induced apoptosis. However, further research is needed, first to determine whether PKCOS affects ATP production in terms of glycolytic pathways and energy involvement in cell proliferation and then to determine the appropriate supplemental dose for gut health and disease prevention in pigs.

**Figure 8:**
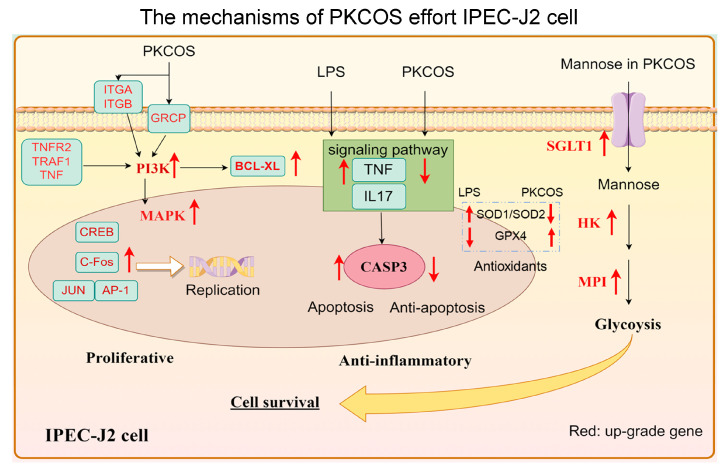
Effect of PKCOS on the survival rate of IPEC-J2 cells with or without LPS treatment. Red arrows up represent gene upregulation and red arrows down represent gene downregulation.

Overall, these findings suggest that the potential roles of PKCOS can be summarised as promoting cell proliferation and alleviating oxidative stress and apoptosis caused by LPS (Figure 8). IPEC-J2 cell proliferation was promoted at a concentration of 1000 µg/mL PKCOS through the upregulated expression of *TNF-α*, *PI3KAP1*, *MAP3K5* and *Fos* in PI3K/AMPK signalling pathways, while cell viability was damaged at a concentration of 5 µg/mL LPS. PKCOS alleviated the cell apoptosis induced by LPS through downregulating expressions of target genes associated with cell death, such as *CASP3* and *TNF-α*, and regulated the expression of antioxidant genes, such as *SOD1*, *SOD2* and *GPX4*, to exert positive antioxidant effects. PKCOS may enter cells through the transporter SGLT1 and promote the glycolytic pathway associated with cell proliferation and the alleviation of inflammation-induced apoptosis.

However, although some achievements were obtained, the present study has limitations that should be further investigated. For example, the antioxidant and anti-inflammatory properties could be attributed to other components, such as flavones. Furthermore, the key regulatory molecules of PKCOS affecting IPEC-J2 cells were not subjected to additional functional validation, such as protein expression or direct observation by immunofluorescence (other than qPCR).

## 5. Conclusions

In this study, an extract (PKCOS) from pre-treated PKCs was obtained, and it largely contained sugar and mannose, plus a very small number of polyphenols and flavones. In vitro studies have demonstrated that PKCOS has antioxidant activity and regulates the expression of antioxidant genes in IPEC-J2 cells. Furthermore, PKCOS has been shown to promote cell proliferation through the activation of the PI3K/MAPK signalling pathway and to regulate the expression of key target genes to alleviate LPS-induced apoptosis. The process of PKCOS-regulated cell survival may be related to glycolysis. This study provides a new approach for the exploitation of derivatives from fermented feeds as antioxidants and prebiotics.

## Figures and Tables

**Table 2:** The contents of PKCOS. The data are expressed as mean ± SD; *n* = 3.

Items	PKCOS
Freeze-dried powder, mg/g PKC	96.19 ± 2.21
PKCOS acquisition rate, %	9.62 ± 0.22
Sugar, % in powder	60.75 ± 0.45
Mannose, % in powder	36.80 ± 0.58
Protein, % in powder	0.19 ± 0.005
Polyphenols, gallic acid equivalents, mg/g PKCOS	17.30 ± 0.02
Flavone, rutin equivalent, mg/g PKCOS	5.90 ± 0.01

## Data Availability

Data are contained within the article.
